# Supplementary material for: Dynalign II: common secondary structure prediction for RNA homologs with domain insertions
Source: Nucleic Acids Res. 2014 Nov 21;42(22):13939–48. doi: 10.1093/nar/gku1172 (PMC4267632; doi:10.1093/nar/gku1172)
Supplement: SUPPLEMENTARY DATA [file supp_gku1172_nar-02021-z-2014-File012.zip › manual/Overview/Interfaces_Summary.html]

RNAstructure Installation and Overview: Interfaces Summary


|  |  |  |
| --- | --- | --- |
|  | RNAstructure Installation and Overview Interfaces Summary | - Contents - Index |
| Name: \| AllSub; Type: \| Text Interface; Location: \| RNAstructure/AllSub; Description: \| Generate a group of suboptimal structures.; To Build: \| In the RNAstructure/ directory, type "make AllSub." --- Name: \| bifold; Type: \| Text Interface; Location: \| RNAstructure/bifold; Description: \| Do bimolecular secondary structure prediction by free energy minimization, allowing intramolecular pairs.; To Build: \| In the RNAstructure/ directory, type "make bifold." --- Name: \| bipartition; Type: \| Text Interface; Location: \| RNAstructure/bipartition; Description: \| Do a bimolecular partition function with two sequences, not allowing intramolecular pairs.; To Build: \| In the RNAstructure/ directory, type "make bipartition." --- Name: \| CircleCompare; Type: \| Text Interface; Location: \| RNAstructure/CircleCompare; Description: \| Compare two structures by creating a postscript file, overlaying their backbones on a circle.; To Build: \| In the RNAstructure/ directory, type "make CircleCompare." --- Name: \| ct2dot; Type: \| Text Interface; Location: \| RNAstructure/ct2dot; Description: \| Convert a CT structure file to a dot bracket structure file.; To Build: \| In the RNAstructure/ directory, type "make ct2dot." --- Name: \| dot2ct; Type: \| Text Interface; Location: \| RNAstructure/dot2ct; Description: \| Convert a dot bracket structure file to a CT structure file.; To Build: \| In the RNAstructure/ directory, type "make dot2ct." --- Name: \| draw; Type: \| Text Interface; Location: \| RNAstructure/draw; Description: \| Draw a Postscript image of a structure.; To Build: \| In the RNAstructure/ directory, type "make draw." --- Name: \| DuplexFold; Type: \| Text Interface; Location: \| RNAstructure/DuplexFold; Description: \| Do bimolecular secondary structure prediction by free energy minimization, with intramolecular pairs forbidden.; To Build: \| In the RNAstructure/ directory, type "make DuplexFold." --- Name: \| dynalign; Type: \| Text Interface; Location: \| RNAstructure/dynalign; Description: \| Find the common secondary structure for two sequences.; To Build: \| In the RNAstructure/ directory, type "make dynalign." --- Name: \| dynalign-smp; Type: \| Text Interface; Location: \| RNAstructure/dynalign; Description: \| Find the common secondary structure for two sequences. This program can be run in a parallel SMP environment.; To Build: \| In the RNAstructure/ directory, type "make dynalign-smp." --- Name: \| DynalignDotPlot; Type: \| Text Interface; Location: \| RNAstructure/DotPlots; Description: \| Create a Postscript dot plot of pairing energies from a Dynalign save file.; To Build: \| In the RNAstructure/ directory, type "make DynalignDotPlot." --- Name: \| efn2; Type: \| Text Interface; Location: \| RNAstructure/efn2; Description: \| Calculate the folding free energy change of structures.; To Build: \| In the RNAstructure/ directory, type "make efn2." --- Name: \| EnergyPlot; Type: \| Text Interface; Location: \| RNAstructure/DotPlots; Description: \| Create a Postscript dot plot of pairing energies from a folding save file.; To Build: \| In the RNAstructure/ directory, type "make EnergyPlot." --- Name: \| EnsembleEnergy; Type: \| Text Interface; Location: \| RNAstructure/EnsembleEnergy; Description: \| Calculate the ensemble folding free energy change for a sequence.; To Build: \| In the RNAstructure/ directory, type "make EnsembleEnergy." --- Name: \| Fold; Type: \| Text Interface; Location: \| RNAstructure/fold; Description: \| Fold a single sequence into its lowest free energy conformation.; To Build: \| In the RNAstructure/ directory, type "make Fold." --- Name: \| Java Cross-Platform Interface; Type: \| GUI Interface; Location: \| RNAstructure/RNAstructure\_java\_interface; Description: \| A cross-platform GUI for RNAstructure.; To Build: \| In the RNAstructure/RNAstructure\_java\_interface directory, type "make." --- Name: \| MaxExpect; Type: \| Text Interface; Location: \| RNAstructure/MaxExpect; Description: \| Predict maximum expected accuracy structures.; To Build: \| In the RNAstructure/ directory, type "make MaxExpect." --- Name: \| multilign; Type: \| Text Interface; Location: \| RNAstructure/multilign; Description: \| Find the structures common for more than two homologous sequences.; To Build: \| In the RNAstructure/ directory, type "make multilign." --- Name: \| multilign-smp; Type: \| Text Interface; Location: \| RNAstructure/multilign; Description: \| Find the structures common for more than two homologous sequences. This program can be run in a parallel SMP environment.; To Build: \| In the RNAstructure/ directory, type "make multilign-smp." --- Name: \| NAPSS; Type: \| Text Interface; Location: \| RNAstructure/napss; Description: \| Use NMR data to assist in predicting secondary structure.; To Build: \| In the RNAstructure/ directory, type "make NAPSS." --- Name: \| oligoscreen; Type: \| Text Interface; Location: \| RNAstructure/oligoscreen; Description: \| Calculate thermodynamic properties of a set of oligonucleotides.; To Build: \| In the RNAstructure/ directory, type "make oligoscreen." --- Name: \| OligoWalk; Type: \| Text Interface; Location: \| RNAstructure/OligoWalk; Description: \| Calculate affinity of target RNA binding by oligomers complementary to a specified RNA target.; To Build: \| In the RNAstructure/ directory, type "make OligoWalk." --- Name: \| partition; Type: \| Text Interface; Location: \| RNAstructure/pfunction; Description: \| Calculate the partition function of a sequence.; To Build: \| In the RNAstructure/ directory, type "make partition." --- Name: \| PARTS; Type: \| Text Interface; Location: \| RNAstructure/PARTS; Description: \| Calculate common structures and pairing probabilities for two unaligned sequences.; To Build: \| In the RNAstructure/ directory, type "make PARTS." --- Name: \| ProbabilityPlot; Type: \| Text Interface; Location: \| RNAstructure/DotPlots; Description: \| Create a Postscript dot plot of base pair probabilities from a partition function save file.; To Build: \| In the RNAstructure/ directory, type "make ProbabilityPlot." --- Name: \| ProbablePair; Type: \| Text Interface; Location: \| RNAstructure/ProbablePair; Description: \| Output structures composed of pairs with pairing probabilities that exceed one or more specific probability thresholds.; To Build: \| In the RNAstructure/ directory, type "make ProbablePair." --- Name: \| ProbKnot; Type: \| Text Interface; Location: \| RNAstructure/ProbKnot; Description: \| Predict secndary structures that may contain pseudoknots.; To Build: \| In the RNAstructure/ directory, type "make ProbKnot." --- Name: \| refold; Type: \| Text Interface; Location: \| RNAstructure/refold; Description: \| Refold a previously folded sequence.; To Build: \| In the RNAstructure/ directory, type "make refold." --- Name: \| RemovePseudoknots; Type: \| Text Interface; Location: \| RNAstructure/RemovePseudoknots; Description: \| Remove as few pairs as possible to transform a pseudoknotted structure into a non-pseudoknotted one.; To Build: \| In the RNAstructure/ directory, type "make RemovePseudoknots." --- Name: \| scorer; Type: \| Text Interface; Location: \| RNAstructure/scorer; Description: \| Score a predicted structure against an accepted one.; To Build: \| In the RNAstructure/ directory, type "make scorer." --- Name: \| stochastic; Type: \| Text Interface; Location: \| RNAstructure/stochastic; Description: \| Sample structures from the Boltzmann ensemble.; To Build: \| In the RNAstructure/ directory, type "make stochastic." --- Name: \| TurboFold; Type: \| Text Interface; Location: \| RNAstructure/TurboFold; Description: \| Find the structures common for more than two homologous sequences, using base pairing probabilities.; To Build: \| In the RNAstructure/ directory, type "make TurboFold." --- Name: \| TurboFold-smp; Type: \| Text Interface; Location: \| RNAstructure/TurboFold; Description: \| Find the structures common for more than two homologous sequences, using base pairing probabilities. This program can be run in a parallel SMP environment.; To Build: \| In the RNAstructure/ directory, type "make TurboFold-smp." --- Name: \| Windows Native Interface; Type: \| GUI Interface; Location: \| RNAstructure/RNAstructure\_windows\_interface; Description: \| A natively compiled Windows GUI for RNAstructure.; To Build: \| TODO? --- Name: \| Windows Text Interfaces; Type: \| Text Interface; Location: \| RNAstructure/RNAstructure\_windows\_text\_interfaces; Description: \| Natively compiled Windows text interfaces for RNAstructure.; To Build: \| TODO? | | |
| Visit The Mathews Lab RNAstructure Page for updates and latest information. | | |
